# Drug-Eluting Balloons in Calcified Coronary Lesions: A Meta-Analysis of Clinical and Angiographic Outcomes

**DOI:** 10.3390/jcm13102779

**Published:** 2024-05-09

**Authors:** Borja Rivero-Santana, Alfonso Jurado-Roman, Guillermo Galeote, Santiago Jimenez-Valero, Ariana Gonzalvez, Daniel Tebar, Raul Moreno

**Affiliations:** 1 Cardiology Department, La Paz University Hospital, 28046 Madrid, Spain; ggaleote1@gmail.com (G.G.); sjvcardio@yahoo.es (S.J.-V.); arianagonzalvez@gmail.com (A.G.); daniel.tebar.m@gmail.com (D.T.); raulmorenog@hotmail.com (R.M.); 2 Hospital La Paz Institute for Health Research (IdiPAZ), 28040 Madrid, Spain

**Keywords:** drug-eluting balloon, calcified coronary lesions, coronary artery disease, complex coronary interventions, drug-eluting stent

## Abstract

**Background:** The usefulness of drug-eluting balloons (DEBs) has not been fully elucidated in calcified coronary lesions (CCLs). This meta-analysis aimed to evaluate the efficacy of DEBs compared to a drug-eluting stent (DES) in this setting. **Methods:** PubMed, EMBASE and Cochrane were searched through December 2023. The primary endpoint was 12 months major adverse cardiac events (MACE). Secondary endpoints included clinical outcomes and angiographic results after PCI and at a 12-month follow-up. **Results:** Five studies and a total of 1141 patients with 1176 coronary lesions were included. Overall, the DEB was comparable to DES in MACE (RR = 0.86, 95% CI: 0.62–1.19, *p* = 0.36), cardiac death (RR = 0.59, 95% CI: 0.23–1.53, *p* = 0.28), myocardial infarction (RR = 0.89, 95% CI: 0.25–3.24, *p* = 0.87) and target lesion revascularization (RR = 1.1, 95% CI: 0.68–1.77, *p* = 0.70). Although the DEB was associated with worse acute angiographic outcomes (acute gain; MD = −0.65, 95% CI: −0.73, −0.56 and minimal lumen diameter; MD = −0.75, 95% CI: −0.89, −0.61), it showed better results at 12 months follow-up (late lumen loss; MD = −0.34, 95% CI: −0.62, −0.07). **Conclusions:** This meta-analysis showed that the DEB strategy is comparable to DES in the treatment of CCLs in terms of clinical outcomes. Although the DEB strategy had inferior acute angiographic results, it may offer better angiographic results at follow-up.

## 1. Introduction

Calcified coronary lesions (CCLs) are present in at least one-third of patients undergoing percutaneous coronary intervention (PCI) [1,2,3,4]. Nevertheless, despite improvements in interventional techniques, PCI in calcified lesions remains a challenge. A CCL could increase technical difficulties, and it is associated with worse acute outcomes due to multiple anatomical and technical factors [5,6] and has been shown to be a predictor of long-term adverse events, including death [7,8,9,10,11].

Nowadays, PCI with drug-eluting stent (DES) implantation represents the most common revascularization strategy in this setting. Although the results of a DES have improved over the last decade, they remain suboptimal in calcified lesions. Several reasons that may explain these findings include the limitation of DES lesion crossing, altered drug elution kinetics and interference with optimal stent expansion [12,13]. Therefore, the development and application of new strategies in CCLs are essential to improve the safety and efficacy of PCI. In recent years, the stent-less drug-coated balloon (DEB) strategy has been shown to be an effective alternative in a variety of scenarios such as in-stent restenosis (ISR) [14,15] and small vessel disease [16,17]. A stent-less DEB strategy has the advantage that it may avoid the need for stent implantation, which could be particularly useful for well-prepared calcified plaques.

However, the potential DEB benefits in the treatment of CCLs have not been well studied. Although some observational studies have shown the feasibility of DEBs in this setting, there are currently no randomized controlled trials comparing results between DESs and DEBs. For this reason, our study aims to address this issue by performing a meta-analysis to assess the current situation and compare the outcomes of the stent-less DEB strategy with the conventional DES strategy for the treatment of CCLs.

## 2. Materials and Methods

### 2.1. Literature Sources and Search Strategies

This meta-analysis was conducted according to the Preferred Reporting Items for Systematic Reviews (PRISMA) guidelines. A systematic and comprehensive literature search using PubMed, Embase and Cochrane Library electronic databases was conducted for studies reporting the drug-coated balloon treatment of calcified coronary lesions from the date of inception to December 2023. The following search terms were used in various combinations: drug-coated balloon, drug-eluting balloon, paclitaxel-eluting balloon, sirolimus-eluting balloon, paclitaxel-coated balloon, sirolimus-coated balloon, calcified coronary arteries, coronary artery calcification, calcification and calcified. No language or sample size restriction was enforced.

**Figure 1:**
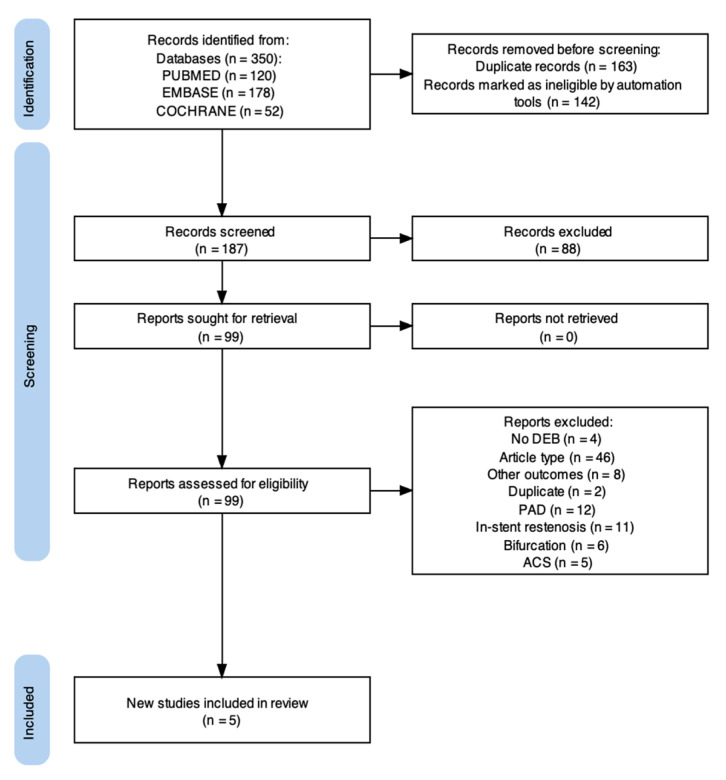
The Preferred Reporting Items for Systematic Reviews and Meta-Analyses (PRISMA) flow diagram describes each phase of the selection process of studies included in the meta-analysis.

The search strategy was carried out by two investigators (B.R.S and A.J.R) who screened the titles and abstracts. All results were exported to EndNote 20 (Clarivate, Philadelphia, PA, USA), and obvious duplicates were removed. The full text of the relevant literature was screened, and eligible trials were selected according to the inclusion and exclusion criteria. Papers without full text were excluded. If a consensus could not be reached between the two authors, the discrepancy was resolved by a third senior author (R.M). In addition to data on the outcomes of interest, a summary of study characteristics, patient characteristics and treatment information was collected. Figure 1 shows the PRISMA literature search flow diagram.

### 2.2. Selection Criteria and Study Outcomes

Using the Population, Interventions, Comparison, Outcomes, and Study Design (PICOS) strategy, studies were included if they met the following criteria: (1) patients aged  ≥  18 years old with moderate-to-severe calcified coronary lesions, defined as radiopacities noted without cardiac motion before contrast injection and generally involving both sides of the arterial wall; (2) there was an intervention group undergoing DEB angioplasty (3); comparing with stent implantation; (4) reporting data on any of the outcomes of interest (MACE, MI, TLR, acute gain, MLD, LLL) and (5) studies were observational in nature. The exclusion criteria included (1) studies involving the use of DEBs in restenosis; (2) small vessel lesions; (3) non-calcified de novo lesions or (4) myocardial infarction. Additionally, studies without reliable data, case reports and review articles were excluded.

The primary endpoint was a composite of major adverse cardiac events (MACE) at 12 months. Secondary outcomes of interest included cardiac mortality, myocardial infarction (MI), target lesion revascularization (TLR), late lumen loss (LLL) at 12 months and post-procedural acute gain (AG) and minimal lumen diameter (MLD). Myocardial infarction was defined according to the universal classification [18]. TLR was defined as revascularization for lesions following stent implantation or DEB angioplasty within the stent or dilated segment or within the 5 mm borders adjacent to the stent or dilated segment. AG was defined as the difference between pre-procedural and post-PCI minimal lumen diameter. LLL was defined as the difference between the diameter of a treatment segment after the procedure compared to the follow-up angiogram.

### 2.3. Data Extraction and Risk of Bias Evaluation

We extracted study information, such as the study design, number of patients, major endpoint, type of DEB, reference vessel diameter, plaque preparation strategy, follow-up and other related information, from the selected studies, and then summarized it in Table 1.

**Figure 2:**
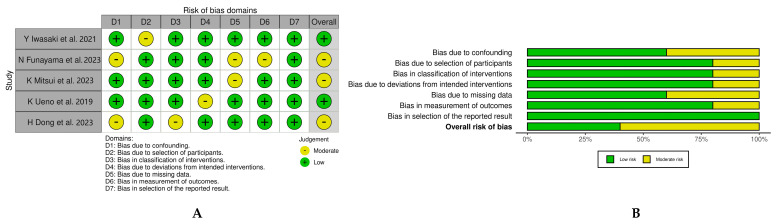
Risk of bias assessment using ROBINS−I tool according to Cochrane network. Risk of bias summary (**A**) and risk of bias graph (**B**) [19,20,21,22,23].

The Risk of Bias in Non-Randomized Studies of Interventions (ROBINS-I) tool was systematically used to assess the five included studies for the risk of bias according to the guidelines outlined in the Cochrane Handbook for Systematic Reviews of Interventions. The assessment focused on several key domains: the process of participant selection, the classification of interventions, deviations from intended interventions, missing data, the measurement of outcomes and the selection of reported results. Studies were evaluated using the risk labels ‘low risk of bias’, ‘moderate risk of bias’, ‘serious risk of bias’ and ‘critical risk of bias’, as depicted in Figure 2. Two independent reviewers (B.R.S and A.J.R) assessed the risk for bias. When there was disagreement, a third reviewer checked the data and made the final decision (R.M).

### 2.4. Statistical Analysis

The effect size was estimated as risk ratios with 95% confidence intervals (CIs) for dichotomous outcomes and as mean difference with the 95% CI for continuous outcomes. The inverse variance method was used in both cases. The I^2^ statistic was applied to judge the degree of heterogeneity among the studies. I^2^ > 40% indicated the presence of heterogeneity in the relevant statistics, and in this case, a random effects model was used. Otherwise, a fixed effects model was used. Publication bias with respect to the outcomes was assessed using a funnel plot and as well with the adjusted rank correlation test according to the method of Begg and Mazumdar. Sensitivity analysis was performed excluding each study separately to assess their effect on observed outcomes. Results with an overall alpha level of 0.05 were considered for statistical significance. All analyses were performed using RevMan 5.4 (Cochrane Collaboration, 2020) and R Project 4.2.1.3.

## 3. Results

### 3.1. Eligible Studies

The search strategy identified 350 articles. Five studies met the inclusion criteria and were included in the analysis [19,20,21,22,23]. Among the included articles, four were prospective cohort studies and one was a retrospective cohort study. Overall, 1141 patients with 1176 coronary lesions were included. Among those, 325 lesions were treated with DEBs and 855 lesions with DESs. Study characteristics are presented in Table 1. A single brand of a paclitaxel-coated balloon (SeQuent Please, B. Braun, Melsungen, Germany) was used as the DEB in all studies. For the control group, stenting was performed using second- or third-generation DESs.

### 3.2. Primary Endpoint

**Figure 3:**
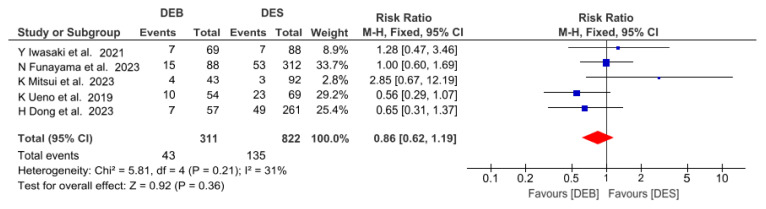
Forest plots of MACE in patients undergoing drug−eluting balloon angioplasty versus drug-eluting stent implantation in calcified coronary lesions. The size of the data marker is proportional to the weight of the individual studies, measured as the inverse of the variance [19,20,21,22,23].

MACE data at 12 months were available for all patients in the five studies included in the analysis. The definition of MACE was homogeneous across studies, although in the article from H Dong et al. [23], stroke was included in the composite of major events. The risk ratio for this primary endpoint was numerically lower for DEBs as compared with the DES group, but this difference was not statistically significant for DEBs compared with the DES group (RR 0.86 [0.62 to 1.19]; *p* = 0.36; I^2^ = 31%) (Figure 3). Sensitivity analyses adding or removing studies with large samples did not change the overall risk of MACE.

### 3.3. Secondary Clinical Endpoints

**Figure 4:**
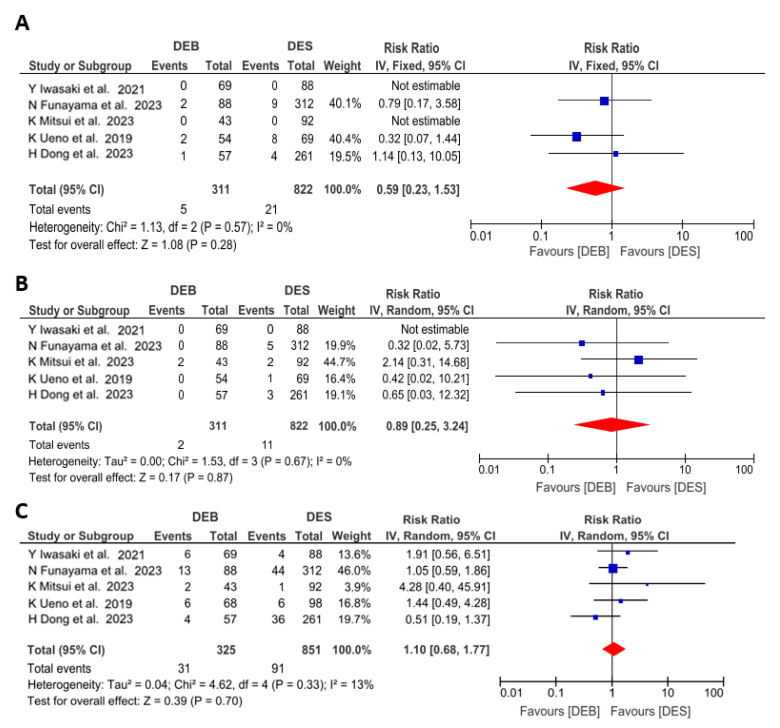
Risk ratios of cardiac death (**A**), myocardial infarction (**B**) and target lesion revascularization (**C**) associated with undergoing drug-eluting balloon angioplasty versus drug−eluting stent implantation in calcified coronary lesions. The size of the data marker is proportional to the weight of the individual studies, measured as the inverse of the variance [19,20,21,22,23].

The results of the meta-analysis for the secondary endpoints of cardiac death, MI and TLR at 12 months are illustrated in Figure 4. Cardiac death (RR 0.59 [0.23 to 1.53]; *p* = 0.28; I^2^ = 0%) and MI (RR 0.89 [0.25 to 3.24]; *p* = 0.87; I^2^ = 0%) were numerically lower for DEBs as compared with the DES group, but these differences were not statistically significant. TLR was similar for the DEB group and the DES group (RR 1.1 [0.68 to 1.77]; *p* = 0.70 I^2^ = 13%).

### 3.4. Procedural Outcomes

**Figure 5:**
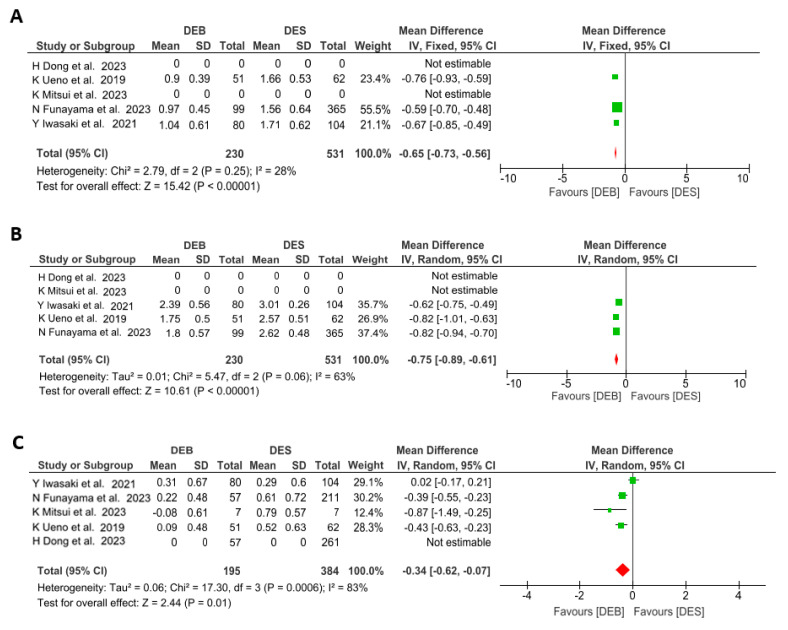
The mean difference of acute gain (**A**), minimal lumen diameter (**B**) and late lumen loss (**C**) associated with undergoing drug−eluting balloon angioplasty versus drug-eluting stent implantation in calcified coronary lesions. The size of the data marker is proportional to the weight of the individual studies, measured as the inverse of the variance [19,20,21,22,23].

Acute angiographic results after the procedure were available in three of the included studies. Worse results in terms of acute gain (MD −0.65 [−0.73 to −0.56]; *p* = < 0.01; I^2^ = 28%) and MLD (MD −0.75 [−0.89 to −0.61]; *p* = 0.06; I^2^ = 63%) were found in the DEB group compared to the DES group, as shown in Figure 5A,B. Data on the angiographic follow-up were available in 579 lesions from four studies. In the pooled estimated mean difference (Figure 5C), LLL was significantly reduced for the DEB group compared with the DES group (MD −0.34 [−0.62 to −0.07]; *p* = 0.01; I^2^ = 83%).

### 3.5. Risk for Bias across Studies

The detailed results of the ROBINS-I risk of bias assessment are presented in Figure 2. We identified a potential risk of bias in the areas of participant selection and intervention classification, which could potentially influence the reported results. Specifically, three out of five studies were assessed as having a moderate risk of bias in the area of participant selection, due to baseline differences between the participants. Although the random effects model used attempts to adjust for this heterogeneity, interpretations of the comparative results between DEBs and DESs should be approached with caution.

## 4. Discussion

This is the first meta-analysis to compare the effectiveness of the stent-less DEB approach against conventional DESs implanted in coronary calcified lesions. The main results of the present study suggest that despite a worse acute angiographic outcome with a stent-less DEB strategy, clinical events are similar, and the DEB was superior in terms of LLL during follow-up.

PCI involving severely calcified lesions remains a challenge in the DES era. Patients with CCL present a higher incidence of cardiac death, myocardial infarction and stent thrombosis at follow-up [24,25]. Several tools alternative to conventional angioplasty balloons have been employed to prepare calcified plaque in the last few years to improve outcomes. These techniques range from rotational atherectomy (RA), orbital atherectomy (OA), intracoronary lithotripsy (IVL) to laser atherectomy (ELCA). However, while these techniques are increasingly used in the routine context, the results after DES implantation are still not optimal in CCL despite their application [26,27,28,29,30]. For this reason, the development of a stent-less DEB strategy may be an attractive option in this clinical scenario. Theoretically, the use of DEBs may have certain advantages over stenting, including a shorter duration of dual antiplatelet therapy, the prevention of neo-atherosclerosis through preserving coronary vasomotion because of the absence of metallic scaffolding implantation and, especially, simplifying procedures in complex anatomies resulting in a reduction in procedural time [31,32,33,34].

The present meta-analysis shows that the event rate is similar after DEB angioplasty and after DES implantation. More specifically, the MACE rate was 13.8% in the DEB group versus 16.4% in the DES group (*p* = 0.36), the cardiac death rate was 1.6% in the DEB group versus 2.5% in the DES group (*p* ≤ 0.0001), MI was 0.6% in the DEB group versus 1.3% in the DES group (*p* = 0.006) and TLR was 9.5% in the DEB group versus 10.7% in the DES group (*p* = 0.70). According to these results, the use of DEBs appears to be a potentially useful alternative in CCLs with the advantage that it could simplify the procedure and avoid stent implantation.

In terms of angiographic outcomes, there were several differences between the DEB and DES strategies. At the time of intervention, the AG was smaller in the stent-less DEB strategy than in the DES group (0.97 ± 0.63 mm versus 1.22 ± 0.62 mm, respectively, *p* < 0.001). Similar results were reported in the MLD (1.99 ± 0.55% in the DEB group versus 2.69 ± 0.44% in the DES group, *p* = 0.06). These differences were to be expected due to the expansion of the lumen at the time of stent placement. However, at the 12-month follow-up, the angiographic results were favorable for the stent-less DEB strategy. In particular, the LLL in the DEB group was significantly lower when compared to the DES group (0.21 ± 0.56 mm versus 0.51 ± 0.68 mm, respectively, *p* = 0.0006). These results are very relevant and should be analyzed. Regarding CCLs, achieving an appropriate AG may be difficult due to the characteristics of the calcified plaque. Furthermore, these suboptimal results have been associated with an increased risk of long-term adverse events [34,35,36]. For this reason, in the setting of patients with CCLs, it is probably worth analyzing the long-term outcome through LLL. This study demonstrates that the LLL is lower in the DEB group. There are several reasons that may explain these results. Potentially, the DES group has worse long-term angiographic outcomes because residual calcium may damage the stent polymer or increase chronic stent recoil, even after lesion preparation with different plaque preparation techniques. In addition, in CCLs, the SeQuent Please drug balloon (B. Braun, Berlin, Germany) can be helpful for drug release because the balloon is folded. In fact, drug washout until reaching the coronary lesion is about 6% [37]. This balloon property allows for the release of most of the drug in the CCL, resulting in potential better long-term outcomes. Even though the studies included in the meta-analysis are not randomized clinical trials, interestingly, the percentage of stent bailout is low among the studies (1.9–8.0%) (Table 1).

This meta-analysis may have a potential impact on clinical practice. The DEB strategy may reduce the intervention time and simplify the treatment of CCLs. Furthermore, we should not forget that CCL patients are often elderly [38]. In this context, DEB therapy may provide an additional benefit by shortening the double aggregation time, especially in these patients who are at high risk of bleeding. Furthermore, because of the favorable results of DEBs in this meta-analysis, a combination of the two approaches, DESs and DEBs, may be possible, especially when other factors (calcified nodules, ISR or small vessel disease) are present.

It is important to note that the results reported in this study were obtained after preparing the calcified plaque with different techniques to the conventional angioplasty balloon (RA/OA). For this reason, the results may not be extrapolated to any CCL. In fact, a stent-less DEB strategy should probably not be applied in the absence of an adequate calcified plaque preparation because of calcified plaque-impeded drug absorption. In addition, only SeQuent Please balloons were included in this study, therefore these results cannot be extrapolated to other DEBs. In fact, there are studies that demonstrate that the effect of different DEBs is not equivalent [39].

## 5. Limitations

The present study has several limitations. First, it is a meta-analysis of cohort studies, and therefore the presence of the selection bias of more favorable lesions is inevitable. However, to date, no clinical trials on DEBs in CCLs have been published. Second, the sample size achieved may be insufficient to demonstrate differences in clinical endpoints. Third, consistent heterogeneity was observed for angiographic outcomes, and a random effects model had to be applied. This heterogeneity, along with the inherent risk of bias in participant selection and intervention classification, necessitates a cautious interpretation of the DEB versus DES comparison. Moreover, the reliance on predominantly observational studies and the potential for bias highlighted by an I^2^ > 40% for MLD and LLL further underscore the need for randomized controlled trials. Fourth, although the majority of the lesions had a reference diameter greater than 2.5 mm, differences in outcomes with respect to small coronary artery disease lesions were not addressed, so a selection bias cannot be excluded. Fifth, the follow-up period was limited to only 12 months, which may be insufficient to detect significant differences in clinical events within this context. Finally, rotational or orbital atherectomy techniques were used in most patients, therefore the results cannot be extrapolated to other patients in cases where other techniques such as conventional balloon angioplasty, IVL or ELCA were applied. However, to our best knowledge, this meta-analysis is the first to compare the use of a stent-less DEB strategy with DESs for CCLs. We believe that longer-term studies or randomized clinical studies with larger sample sizes are needed to confirm these results in the future.

## 6. Conclusions

This meta-analysis suggests that the use of stent-less DEB strategies offers a safety and efficacy profile comparable to DES implantation in the treatment of calcified coronary lesions, especially when followed by adequate plaque preparation. Although the DEB strategy was associated with inferior acute angiographic outcomes, it appears to be associated with better angiographic results at 12 months, with a greater reduction in late lumen loss. While prospective studies with larger sample sizes and a longer follow-up are needed, these findings may support the potential of the DEB technique as a viable alternative in the treatment of CCLs.

## Figures and Tables

**Table 1:** The characteristics of studies included in the meta-analysis. cB: conventional balloon; CB: cutting balloon; DEB: drug-eluting balloon; DES: drug-eluting stent; HBP: high-pressure balloon; MACE: major adverse cardiac event; OA: orbital atherectomy; RA: rotational atherectomy; RVD: reference vessel disease; SB: scoring balloon; TLR: target lesion revascularization.

	Study Design	DEB	Strategies		RVD (mm)		Plaque Preparation Strategy	Follow-Up (Months)	Primary Endpoint	Definition of MACE	Bailout Stenting (%)
			DEB	DES	DEB	DES					
Y Iwasaki et al. [19]	Prospective cohort	SeQuent Please	69	88	3.03 ± 0.36	2.97 ± 0.45	RA	12	MACE	Cardiac death, MI, TLR	8.0
N Funayama et al. [20]	Retrospective cohort	SeQuent Please	88	312	2.42 ± 0.59	2.65 ± 0.59	RA	12	TLR	Cardiac death, MI, TLR	-
K Mitsui et al. [21]	Prospective cohort	SeQuent Please	43	92	3.00 (2.7; 3.50)	3.25 (2.9; 3.50)	OA; cB; CB; SB; HPB	12	MACE	Cardiac death, MI, TLR	2.3
K Ueno et al., 2019 [22]	Prospective cohort	SeQuent Please	54	69	2.28 ± 0.58	2.49 ± 0.55	RA	12, 24, 36	TLR	Cardiac death, MI, TLR	1.9
H Dong et al. [23]	Prospective cohort	SeQuent Please	57	261	2.99 ± 0.34	2.68 ± 0.38	RA	12	MACE	Cardiac death, MI, stroke, TLR	3.5

## Data Availability

The data presented in this study are available on request from the corresponding author.

## Abbreviations

AG	Acute gain
CCL	Calcified coronary lesion
DEB	Drug-eluting balloon
DES	Drug-eluting stent
ELCA	Excimer laser coronary atherectomy
IVL	Intravascular lithotripsy
LLL	Late lumen loss
MACE	Major adverse cardiac events
MI	Myocardial infarction
MLD	Minimal lumen diameter
OA	Orbital atherectomy
PCI	Percutaneous coronary intervention
PRISMA	Preferred Reporting Items for Systematic Reviews and Meta-Analyses
RA	Rotational atherectomy
TLR	Target lesion revascularization
ISR	In-stent restenosis
